# The relationship between risk of bias criteria, research outcomes, and study sponsorship in a cohort of preclinical thiazolidinedione animal studies: a meta‐analysis

**DOI:** 10.1002/ebm2.5

**Published:** 2015-01-20

**Authors:** M. Abdel‐Sattar, D. Krauth, A. Anglemyer, L. Bero

**Affiliations:** ^1^ Department of Clinical PharmacyUniversity of CaliforniaSan FranciscoCAUSA; ^2^ Institute for Health Policy StudiesUniversity of CaliforniaSan FranciscoCAUSA; ^3^ Charles Perkins Centre and Faculty of PharmacyThe University of SydneySydneyNSWAustralia

**Keywords:** bias, meta‐analysis, conflict of interest, preclinical

## Abstract

**Introduction:**

There is little evidence regarding the influence of conflicts of interest on preclinical research. This study examines whether industry sponsorship is associated with increased risks of bias and/or effect sizes of outcomes in published preclinical thiazolidinedione (TZD) studies.

**Methods:**

We identified preclinical TZD studies published between January 1, 1965, and November 14, 2012. Coders independently extracted information on study design criteria aimed at reducing bias, results for all relevant outcomes, sponsorship source and investigator financial ties from the 112 studies meeting the inclusion criteria. The average standardized mean difference (SMD) across studies was calculated for plasma glucose (efficacy outcome) and weight gain (harm outcome). In subgroup analyses, TZD outcomes were assessed by sponsorship source and risk of bias criteria.

**Results:**

Seven studies were funded by industry alone, 17 studies funded by both industry and non‐industry, 49 studies funded by non‐industry alone and 39 studies had no disclosures. None of the studies used sample size calculations, intention‐to‐treat analyses, blinding of investigators or concealment of allocation. Most studies reported favourable results (88 of 112) and conclusions (95 of 112) supporting TZD use. Efficacy estimates were significantly larger in six studies sponsored by industry alone (−3.41; 95% CI −5.21, −1.53; I^2^ = 93%) versus 42 studies sponsored by non‐industry sources (−0.97; 95% CI −1.37, −0.56; I^2^ = 81%; p‐value = 0.01). Harms estimates were significantly larger in four studies sponsored by industry alone (5.00; 95% CI 1.22, 8.77; I^2^ = 93%) versus 38 studies sponsored by non‐industry sources (0.30; 95% CI −0.08, 0.68; I^2^ = 79%; p‐value = 0.02). TZD efficacy and harms did not differ by disclosure of financial COIs or risks of bias.

**Conclusions:**

Industry‐sponsored TZD animal studies have exaggerated efficacy and harms outcomes compared with studies funded by non‐industry sources. There was poor reporting of COIs.

## Introduction

Medications play an essential role in the treatment of disease, but often have harmful side effects that may put patients at risk. The safety and efficacy profiles of medications approved by the U.S. Food and Drug Administration (FDA) are based on data from preclinical animal studies and clinical studies in humans. Drug research has been increasingly funded by pharmaceutical companies over the past few decades.^1^ This has allowed for conflicts of interest (COIs) to arise between researchers and their funders and has made research findings vulnerable to a number of methodological biases. When clinical guidelines and healthcare decisions are based on drug studies with biased research outcomes, patients may receive suboptimal medication therapies and/or suffer from serious adverse effects that could have been otherwise avoided or at least better monitored.

Knowing that various types of bias can be found in human clinical studies funded by pharmaceutical companies,^2^, ^3^ it is reasonable to suspect that industry‐sponsored preclinical animal studies would also have a high potential for bias. However, little is known about the level of bias that may be found in the design of preclinical animal studies, as previous investigations have been limited to case studies documenting discrepancies between industry‐ and government‐sponsored animal researches.^4^, ^5^ The Institute of Medicine (IOM) 2010 report on Conflicts of Interest in Medical Research, Education and Practice highlights the need for systematic reviews to reveal the extent of financial relationships and their consequences in preclinical research.^6^ One systematic review has found that, in contrast to clinical studies, industry‐sponsored preclinical studies underestimate effect sizes of the drugs being tested compared with non‐industry‐sponsored studies.^7^ Thus, industry sponsors may have different incentives that could influence the outcomes of clinical versus preclinical studies.^8^ Further research is needed to identify any consistent biases associated with industry sponsorship of animal studies.

Risks of bias are methodological criteria of a study that can introduce a systematic error in the magnitude or direction of the results. Risk of bias criteria empirically identified in human clinical research, as well as animal experiments,^9^ include randomization, concealment of allocation, blinding of investigators, accounting for all animals, sample size calculations, intention‐to‐treat analyses and animal inclusion/exclusion criteria. The objective of this study is to determine whether industry‐sponsored preclinical trials are more likely to have different efficacy and/or harm estimates compared to non‐industry‐sponsored trials, even when controlling for these risk of bias criteria.

This systematic review focuses on animal studies of thiazolidinediones (TZDs), also known as glitazones, intended for the management of type II diabetes. These oral hypoglycaemic agents were targeted because the market for these drugs is competitive and the vast majority of their safety and efficacy studies are funded by industry. Furthermore, previous research has identified the factors associated with biased results and conclusions of human trials for TZDs.^10^ We hypothesize that industry‐sponsored TZD animal studies will have different efficacy and/or harm estimates compared with non‐industry‐sponsored trials, regardless of their risk of bias.

## Methods

### identification of studies

The selection criteria for studies, data extraction and analyses were all determined prior to data collection. This research was exempt from Institutional Review Board review because it does not involve human subjects.

#### Search strategy

The Medline^®^ database was searched from January 1, 1965, to November 14, 2012, for all published TZD animal studies that compared a TZD to another drug or placebo and reported outcomes of plasma glucose, weight gain and/or other diabetes‐related measures. We included studies of marketed TZDs (e.g. rosiglitazone, pioglitazone, troglitazone) and investigational TZDs (e.g. ciglitazone, netoglitazone, CP 68722).

An expert librarian (GW) was consulted to develop a search strategy containing the following MeSH terms, text words and word variants:

(thiazolidinedione OR glitazone OR rosiglitazone OR pioglitazone OR troglitazone OR rivoglitazone OR ciglitazone) AND (animal* OR preclinical OR “pre‐clinical” OR mice OR rats OR rabbits OR dog OR dogs OR monkey OR monkeys OR “animal experimentation”[MeSH Terms] OR “models, animal”[MeSH Terms] OR “invertebrates”[MeSH Terms] OR “Animals”[MH] OR “animal population groups”[MeSH Terms]) NOT (humans[mh] NOT animals[mh:noexp]) AND (health effect OR health effects OR toxic OR toxicity OR toxicities OR efficacy OR efficacies OR toxicology OR safety OR harm* OR drug effects[sh] OR therapeutic use[sh:noexp] OR adverse effects[sh] OR poisoning[sh] OR pharmacology[sh:noexp] OR chemically induced[sh]) AND eng[la] NOT review[pt] NOT systematic review* NOT meta‐analysis[pt] NOT randomized controlled trial[pt]

#### Inclusion/exclusion criteria

One investigator (DK) screened abstracts and full‐texts from our Medline search to identify the 112 studies meeting the inclusion and exclusion criteria. Included studies had to (1) be published between January 1, 1965, and November 14, 2012, (2) contain results for plasma glucose, weight gain and/or other diabetes‐related measures, (3) have an intervention group receiving only the TZD and (4) compare the TZD with placebo and/or an active comparator. We excluded (1) pharmacokinetic, pharmacodynamic and mechanism of action studies, (2) review articles, systematic reviews, meta‐analyses, editorials, letters‐to‐the‐editor and commentaries, (3) studies with reproductive health outcomes where mothers are treated, (4) studies reporting only on effects of metabolites or derivatives of TZDs and (5) articles with no English translation available.

### data collection and analysis

Three coders (MAS, DK and CG) received training to use the data extraction and quality assessment instrument that was developed for this systematic review. This instrument was modelled after previous studies that followed a similar protocol^10^, ^11^, ^12^, ^13^, ^14^ and included a coding manual. Methodological criteria were based on a published systematic review of tools for assessing biases in animal studies.^9^

Data were extracted into an Excel database. Articles in the database were randomized using the Excel “RAND” function and assessed in random order by the coders. Discrepancies between the coders were adjudicated by discussion among the investigators. Extracted data and coder assessments for risks of bias, study characteristics and outcomes from articles included in the review are available from the Dryad Digital Repository: http://doi.org/10.5061/dryad.4c2bj.^15^

#### Single‐coded data collection

Single‐coded data collection was limited to the extraction of information that required no judgement by the coder. The following characteristics were collected from each included study by a single coder (DK):

##### Study characteristics

Title of the study, month of publication, year of publication and journal name.

##### Author affiliation

The affiliation(s) of the author(s) was obtained from the study by‐line and classified into (1) industry, if all authors were employed by industry, (2) non‐industry, if no author was employed by industry or (3) combined if at least one author was employed by industry and at least one author was not employed by industry. If a single author had affiliations with industry and non‐industry sources, the study was coded as “combined”.

##### Sponsorship source

The source of sponsorship for each study was categorized as (1) any industry, (2) non‐industry, (3) no sponsorship and (4) no sponsorship statement. For studies with disclosed sponsorship, we determined if there was a statement about the role of the sponsor.

##### Financial ties of authors

Information about disclosed financial ties was coded as (1) at least one author of the study reported having a financial conflict of interest, (2) all authors reported having no conflicts of interest and (3) there was no disclosure statement.

##### Study design characteristics

For each study, the following study design criteria were collected: (1) name of TZD used in the study, (2) the comparison groups (e.g. comparator TZD, active comparator non‐TZD drug or placebo), (3) animal species and strain used in the study, (4) number of control and treated animals at the start of the study and (5) whether the study reported morbidity and mortality data or only surrogate outcomes of efficacy and harms based on laboratory analyses.

#### Double‐coded data collection

All risk of bias criteria were coded as (1) yes, if the criterion was met, (2) no, if the criterion was not met and when applicable (3) partial, if the criterion was partially met. Since a level of judgement by coders was required in this process, the following criteria were independently assessed by two coders for each publication:

##### Randomization

*Was the treatment randomly allocated to animal subjects so that each subject has an equal likelihood of receiving the intervention?* Randomization was coded as (1) yes, (2) no and (3) partial. A partial rating was assigned to studies where authors mention having randomized animals in their experiments but provide no details on how that randomization was designed or executed.

##### Concealment of allocation

*Were processes used to protect against selection bias by concealing from the investigators how treatment was allocated at the start of the study?* Concealment of allocation was coded as (1) yes, (2) no and (3) partial.

##### Blinding

*Was the investigator(s) involved with performing the experiment, collecting data and assessing the outcome of the experiment unaware of which subjects received the treatment and which did not?* Blinding was coded as (1) yes, (2) no and (3) partial.

##### Inclusion/exclusion criteria

*Were the criteria used for including or excluding subjects specified?* Inclusion/exclusion criteria were coded as (1) yes, (2) no and (3) partial.

##### Test animal description

*Did the author(s) describe in detail the test animal characteristics including, the animal species, strain, sub‐strain, genetic background, age, supplier, sex, weight. At least one of these characteristics must be present for this criterion to be met*. Test animal description was coded as (1) yes, (2) no and (3) partial.

##### Animal environment described

*Did the author(s) adequately describe the housing and husbandry, nutrition, water, temperature, lighting conditions? At least one of these characteristics must be present for this criterion to be met*. Environmental parameters were coded as (1) yes, (2) no and (3) partial.

##### Dose/response model

*Did the authors justify their choice of an appropriate dose–response model given the research question and disease being modelled?* Dose/response model was coded as (1) yes or (2) no.

##### Optimal time window investigated

*Did the investigator provide sufficient time to pass before assessing the outcome? The optimal time window used in animal research should reflect the time needed to see the outcome*. Optimal time window investigated was coded as (1) yes, (2) no and (3) partial.

##### All animals accounted for

*Did the investigator account for attrition bias by detailing when animals were removed from the study and for what reason they were removed?* All animals accounted for was coded as (1) yes, (2) no and (3) partial. A partial rating was given when the number of animals was listed and justified at the beginning and end of some experiments but not others within the same publication.

##### Intention‐to‐treat analysis

*Did the author(s) perform an intention‐to‐treat analysis (ITT)?* ITT was coded as (1) yes, (2) no and (3) partial.

##### Statement of compliance with animal welfare requirements

*Did the author(s) state whether or not they complied with regulatory requirements for the handling and treatment of test animals?* Statement of compliance with animal welfare requirements was coded as (1) yes or (2) no.

##### Sample size calculation

*Did the authors perform a sample size calculation to justify the total number of animals used in the study?* Sample size calculation was coded as (1) yes or (2) no.

#### Coding of primary outcomes

Four data extractors (DK, AA, CL and MAS) recorded results for diabetes‐related outcomes defined *a priori* by the investigators, including plasma glucose as the primary efficacy measure and weight gain as the primary harms measure. If multiple time points were reported, all time points were included in the meta‐analysis as to not assume a primary endpoint or arbitrarily assign an endpoint in the analysis. For each result, the raw data (often derived from tables, graphs, figures, etc.), measure of effect, confidence interval, measure of variability, p‐value and statistical test used were recorded.

Results were categorized as (1) favourable, if the result was statistically significant (p < 0.05) and in the direction of the TZD being more efficacious or less harmful; (2) unfavourable, if the result was not statistically significant (p > 0.05) or significant in the wrong direction (e.g. TZD statistically more harmful than non‐TZD treatment group); (3) neutral, if the TZD was significantly different in the direction favouring the TZD against one control group (e.g. early control) but not significantly different compared to a second control group (e.g. late control).

If an outcome was measured over multiple time points or concentrations, it was categorized as (1) favourable if at least one measurement was in favour of the TZD or (2) unfavourable if there were no measurements in favour of the TZD. For each included result, data were extracted for mean outcome, standard deviation (SD) or standard error (SE), and the number of treated and untreated animals.

#### Statistical analysis

We report the frequencies of each study design criterion and the coding of the results and conclusions by sponsorship source.

To test our hypothesis, we conducted a meta‐analysis of the studies that had analyzable data. For a study to have analyzable data, an author needed to report both a mean value and a measure of dispersion (SE or SD) or provide adequate data so that we could calculate these measures ourselves. Not all studies containing quantitative (numerical) data had analyzable data.

We calculated the effect of TZDs using a standardized mean difference (SMD) for each outcome. Due to the lack of independence of animals between outcomes within studies, we averaged SMDs and variances across outcomes for each study, yielding k average SMDs and variances for k studies. We pooled the data across studies and estimated summary average SMDs using random‐effects models.^16^ Specifically, we estimated the average SMD for each included study and used the inverse variance method to calculate study weights. The inverse variance method assumes that the variance for each study is inversely proportional to its importance; therefore, more weight is given to studies with less variance than studies with greater variance. The SMD null hypothesis (H_o_: estimate = 0) states that there is no difference in the effect of TZD use on body weight or glucose outcomes when compared with a control or placebo. A number less than zero suggests that the TZD reduces body weight or plasma glucose when compared with control or placebo. A number greater than zero suggests that the TZD increases body weight or plasma glucose when compared with the control or placebo.

We examined heterogeneity among the studies using the I^2^ statistic. We interpreted an I^2^ estimate greater than 50% as indicating moderate or high levels of heterogeneity. We anticipated high levels of heterogeneity as previous meta‐analyses of animal studies have found high levels of heterogeneity between studies, potentially resulting from typical, small sample sizes in animal models.^17^

We further investigated the potential causes of heterogeneity by conducting *a priori* subgroup analyses using the χ^2^ statistic with a significance level of 0.10. We performed subgroup analyses by study criteria that we hypothesized would be associated with effect sizes: sponsorship source, financial ties of authors, randomization, stating inclusion/exclusion criteria for animals, accounting for all animals, dose/response model—justification for TZD dose, and optimal time window investigated.

We evaluated differences in pooled effect estimates between declared sponsorship sources by risk of bias criteria to determine if the effect between sponsorship sources differed by specific risks of bias.

## Results

### identification of studies

**Figure 1:**
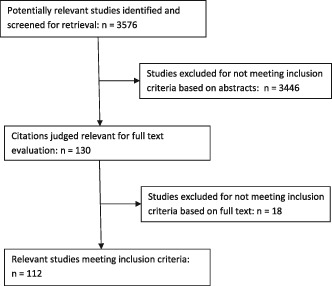
Flowchart for manuscript selection and inclusion. “n” indicates the number of studies.

The initial literature search identified 3,576 articles for review (Figure 1). After screening the abstracts, 130 articles were selected based on the inclusion criteria. Of the 130 publications of interest, 11 were excluded for not being available in English. Seven of the 119 remaining studies were excluded after full text evaluation because they did not have any TZD efficacy or safety data. The final number of studies included was 112.

### data collection and analysis

#### TZDs studied

Among the 112 included articles, the most commonly researched TZDs were rosiglitazone (39.3%), pioglitazone (23.2%) and troglitazone (17.9%). Other TZDs studied (19.6%) included ciglitazone, englitazone, ragaglitazar, chiglitazar, T‐174, mifobate (SR‐202), netoglitazone (MCC‐555) and CP 68,722.

#### Animal species studied

The majority of experiments (92.9%) were conducted in either rats (n = 63) or mice (n = 38), or both (n = 3). Other species studied (7.1%) included monkeys (n = 3), cows (n = 2), horses (n = 1), dogs (n = 1) and rabbits (n = 1).

#### Source of sponsorship

Our cohort of 112 TZD animal studies included 7 studies funded by industry alone, 17 studies funded by both industry and non‐industry sources, 49 studies funded by non‐industry alone and 39 studies with no disclosure of funding source (Table 1). Among the 73 studies with a disclosed sponsor of any type, none stated that the sponsor was directly involved in the study, only 1 explicitly stated that the sponsor was not involved in the study, and 72 did not mention whether the sponsor was involved in the study or not.

**Table 1:** Characteristics of included studies by sponsorship source

Characteristic	Category	Total (n = 112)		Sponsorship source		
				Any industry^a^ (n = 24)	Non‐industry (n = 49)	No disclosure (n = 39)
		n (%)		n (%)	n (%)	n (%)
**Comparison Group**	TZD vs. active comparator drug^b^	53 (47)		8 (33)	24 (49)	21 (54)
	TZD vs. placebo	59 (53)		16 (67)	25 (51)	18 (46)
**Outcome Assessment**	Surrogate Outcomes	111 (99)		24 (100)	48 (98)	39 (100)
	Morbidity	8 (7)		1 (4)	4 (8)	3 (8)
	Mortality	2 (2)		0 (0)	1 (2)	1 (3)
**Risk of Bias** c	Randomization	40 (36)		8 (33)	24 (49)	8 (21)
	Concealment of allocation	0 (0)		0 (0)	0 (0)	0 (0)
	Blinding of investigators	0 (0)		0 (0)	0 (0)	0 (0)
	Inclusion/exclusion criteria	9 (8)		1 (4)	2 (4)	6 (15)
	Sample size calculation	0 (0)		0 (0)	0 (0)	0 (0)
	Test animal description	112 (100)		24 (100)	49 (100)	39 (100)
	Animal environment described	107 (96)		22 (92)	46 (94)	39 (100)
	Dose/response model	22 (20)		5 (21)	10 (20)	7 (18)
	Optimal time window investigated	5 (4)		1 (4)	2 (4)	2 (5)
	All animals accounted for	54 (48)		11 (46)	24 (49)	19 (49)
	Intention‐to‐treat analysis	0 (0)		0 (0)	0 (0)	0 (0)
**Results**	Favours TZD	88 (79)		20 (83)	38 (78)	30 (77)
	Does not favour TZD	7 (6)		1 (4)	5 (10)	1 (3)
	Neutral	17 (15)		3 (13)	6 (12)	8 (21)
**Conclusion**	Favours TZD	95 (85)		21 (88)	38 (78)	36 (92)
	Does not favour TZD	2 (2)		1 (4)	1 (2)	0 (0)
	Neutral	15 (13)		2 (8)	10 (20)	3 (8)
**Conflict of Interest**	Reported conflict	9 (8)		6 (25)	2 (4)	1 (3)
	Reported no conflict	10 (9)		1 (4)	8 (16)	1 (3)
	No disclosure	93 (83)		17 (71)	39 (80)	37 (95)

a The any industry category includes 7 studies sponsored solely by industry and 17 sponsored by industry and non‐industry sources.

b The TZD vs. active comparator category includes 20 studies with active comparators that are not used in the treatment of diabetes (e.g. antihypertensive drugs and anti‐hyperlipidemic drugs).

c Numbers account for studies that fully or partially met the respective risk of bias criteria.

#### Reporting of quality and risk of bias criteria

The most commonly reported methodological criteria were test animal characteristics (100%) and description of the animal environment (95.5%; Table1). These criteria are descriptive in nature and have not been empirically associated with biased research outcomes. The most commonly reported risk of bias criteria were randomization (35.7% of studies) and accounting for all animals (48.2%) in experiments. Only a few studies justified their optimal time window for observing TZD efficacy and/or harms (4.5%), specified inclusion/exclusion criteria (8.0%) and applied a dose/response model to justify the dose of TZD chosen (19.6%). Moreover, none of the studies in our cohort used concealment of allocation, blinding of investigators, sample size calculations or intention‐to‐treat analyses (Table1).

#### Financial ties of authors

Only 8.1% (9 of 112) of studies disclosed that at least one author had financial COIs and 83.0% (93 of 112) of studies did not have a COI disclosure statement (Table1).

#### Reported results and conclusions

Overall, 78.6% (88 of 112) of studies reported quantitative results that favoured TZDs. Similarly, 84.8% (95 of 112) of studies contained author conclusions that favoured TZD use (Table1). Of studies with some industry‐sponsorship, 83.3% (20 of 24) reported favourable results, while 77.6% (38 of 49) of non‐industry‐sponsored studies reported favourable results. The relative risk (RR) of having favourable results when comparing industry‐sponsored studies with studies without industry sponsorship was 1.07 (95% CI 0.85, 1.36).

Similarly, 87.5% of studies (21 of 24) with some industry sponsorship and 77.6% of studies (38 of 49) sponsored by non‐industry sources had conclusions that favoured TZDs. The RR of having favourable conclusions when comparing industry‐sponsored studies with studies without industry sponsorship was 1.13 (95% CI 0.91, 1.40).

#### Reported outcomes

The most commonly reported outcomes in TZD animal studies were plasma glucose (83.9%) and plasma insulin (75%), followed by weight gain (64.3%) and free fatty acids (53.6%).

#### A priori subgroup analyses

**Figure 2:**
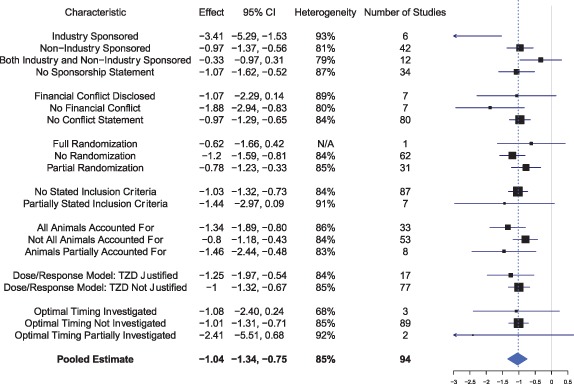
Meta‐analysis of 94 animal studies estimating effect of TZDs on plasma glucose. Horizontal lines indicate 95% confidence intervals (CIs) and squares reflect the point estimate. The diamond reflects the pooled estimate across all studies.

Across 94 studies with analyzable plasma glucose measures, the effect size significantly favoured TZDs (−1.04; 95% CI −1.34, −0.75), with substantial heterogeneity (I^2^ = 85%; Figure 2). The effect of TZDs on plasma glucose was greater in 6 industry‐sponsored studies compared with 42 studies having no industry sponsorship (test for subgroup differences: p = 0.01) and in 6 industry‐sponsored studies compared with 34 studies having no sponsorship statement (test for subgroup differences: p = 0.02; Figure 2).

**Figure 3:**
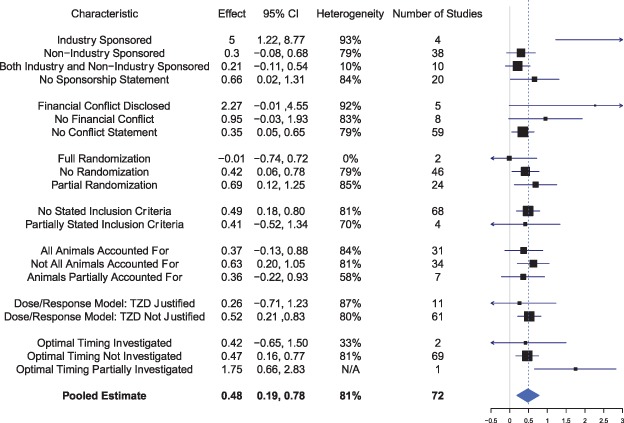
Meta‐analysis of 72 animal studies estimating effect of TZDs on weight gain. Horizontal lines indicate 95% confidence intervals (CIs) and squares reflect the point estimate. The diamond reflects the pooled estimate across all studies.

As for weight gain, a common side effect of TZDs, the effect size based on 72 studies with analyzable harms data was 0.48 (95% CI 0.19, 0.78), with substantial heterogeneity (I^2^ = 81%; Figure 3). The effect of TZDs on body weight gain was greater in 4 industry‐sponsored studies compared with 38 studies with no industry sponsorship (test for subgroup differences: p = 0.02) and in 4 industry‐sponsored studies compared with 20 studies with no sponsorship statement (test for subgroup differences: p = 0.03; Figure 3).

We performed a sensitivity analysis on plasma glucose measures (Figure 2) using more specific sponsorship groupings: industry sponsored (n = 6), combined industry and non‐industry sponsored (n = 12), non‐industry sponsored (n = 42) and no funding statement (n = 34). Results were exaggerated in studies with industry‐sponsorship alone (−3.41; 95% CI −5.29, −1.53; I^2^ = 93%) compared with combined sponsorship (−0.33; 95% CI −0.97, 0.31; I^2^ = 79%), non‐industry sponsorship (−0.97, 95% CI −1.37, −0.56; I^2^ = 81%) and no funding statement (−1.07; 95% CI −1.62, −0.52; I^2^ = 87%). However, grouping studies fully and partially sponsored by industry together (n = 18) did not yield similar results (−1.14; 95% CI −1.82, −0.47; I^2^ = 87%) as demonstrated by a test for subgroup differences between any industry and no industry sponsorship (p = 0.66).

We performed the same sensitivity analysis on weight gain measures (Figure 3) using more specific sponsorship groupings: industry sponsored (n = 4), combined industry and non‐industry sponsored (n = 10), non‐industry sponsored (n = 38) and no funding statement (n = 20). Results were again exaggerated in studies with industry‐sponsorship alone (5.00; 95% CI 1.22, 8.77; I^2^ = 93%) compared with combined sponsorship (0.21; 95% CI −0.11, 0.54; I^2^ = 10%), non‐industry sponsorship (0.30, 95% CI −0.08, 0.68; I^2^ = 79%) and no funding statement (0.66; 95% CI 0.02, 1.31; I^2^ = 84%). However, grouping studies fully and partially sponsored by industry together (n = 14) did not yield similar results (0.78; 95% CI 0.10, 1.47; I^2^ = 81%) as demonstrated by a test for subgroup differences between any industry and no industry sponsorship (p = 0.23).

#### Risks of bias by sources of sponsorship

For both plasma glucose and weight gain measures, none of the risk of bias criteria resulted in remarkably different effect sizes in comparison with pooled estimates (Figures 2 and 3^3^). For example, the estimated TZD effect on blood glucose (Figure 2) did not vary widely between studies where all animals were accounted for (−1.34; 95% CI −1.89, −0.80; I^2^ = 86%), partially accounted for (−1.46; 95% CI −2.44, −0.48; I^2^ = 83%) or not accounted for (−0.80; 95% CI −1.18, −0.43; I^2^ = 84%) as demonstrated by a test for subgroup differences between 33 studies accounting for all animals and 53 studies not doing so (p = 0.11). Similarly, the estimated TZD effect on weight gain (Figure 3) did not vary widely between studies where all animals were accounted for (0.37; 95% CI −0.13, 0.88; I^2^ = 84%), partially accounted for (0.36; 95% CI −0.22, 0.93; I^2^ = 58%), or not accounted for (0.63; 95% CI 0.20, 1.05; I^2^ = 81%) as demonstrated by a test for subgroup differences between 31 studies accounting for all animals and 34 studies not doing so (p = 0.11).

## Discussion

Building upon the evidence that biases are common in human clinical drug studies, including studies of TZDs, funded by pharmaceutical companies,^2^, ^10^ this systematic review investigated bias in the design of TZD preclinical studies and examined the association between industry support and the outcomes. Assessment of the 112 included TZD animal studies showed evidence of poor reporting of risk of bias criteria regardless of sponsorship source, exaggerations in the effect size of efficacy and harms outcomes in industry‐sponsored studies, and non‐disclosure of funding sources (34.8%) or financial ties of investigators (83.0%) in a substantial number of articles.

Owing to the poor reporting of risk of bias criteria, we could not identify differences in risks of bias between the non‐industry and industry‐sponsored studies. None of the studies in our cohort had sample size calculations, intention‐to‐treat analyses, concealment of allocation or blinding of investigators. Descriptive criteria specifying the type of animals used (100%) and their environment (95.5%) were readily available. These descriptive criteria may be better reported because a number of guidelines for publishing animal research require them,^9^ including the Animal Research: Reporting of *In Vivo* Experiments (ARRIVE) guidelines^18^ released in 2010. However, risk of bias criteria, such as randomization and blinding, should be held to the same reporting standard as these descriptive criteria since there is empirical evidence that they affect the outcomes of animal research.^19^, ^20^, ^21^, ^22^, ^23^

In order to gather additional evidence on the association between risks of bias and efficacy or harm effect sizes in future meta‐analyses, better reporting of risk of bias criteria needs to be implemented in animal research. Recent calls for reporting criteria in animal studies recognize the need for the adoption and enforcement of journal reporting standards.^24^, ^25^ In clinical research, reporting of risk of bias criteria improved once investigators began performing risk of bias assessments through systematic reviews and once journals began adopting reporting standards.^26^ Similarly, we expect reporting in animal research to improve if risk of bias assessments become more common.

Our findings confirmed that industry funding of animal research can lead to different effect sizes being reported for both efficacy and harms outcomes compared with non‐industry supported research. The exaggeration of the efficacy estimate, namely plasma glucose, in industry‐sponsored studies suggests that the studies are biased towards reporting more efficacious results. However, this overestimation of efficacy was accompanied with an increase in harms, namely weight gain, in those same industry‐sponsored studies. This contrasts with the findings of underestimation of harms reported in human drug trials sponsored by industry.^2^ Industry‐sponsored studies may test higher doses on more animals for longer periods of time which could potentially enhance efficacy and harm measures. The observed difference in effect size for efficacy and harms needs replication as there was a limited number of studies in our cohort that were sponsored solely by industry (n = 7) and which reported analyzable outcomes for plasma glucose (n = 6) and body weight (n = 4).

A number of studies had conclusions favouring TZDs, regardless of whether the results supported TZDs or not. A previous analysis of preclinical studies of statins found a notable discordance between results and conclusions in industry‐sponsored studies compared with non‐industry‐sponsored studies.^7^ This discrepancy between results and conclusions has also been observed in meta‐analyses of randomized controlled trials and trials of drugs conducted in humans.^2^, ^3^ However, this discordance was less evident in our cohort of TZD animal studies as both industry and non‐industry‐sponsored studies had conclusions that were more favourable to the test drug than the results reported within those same studies.

## Limitations

This systematic review is based on a search strategy limited to articles accessible through the Medline database and available in English. Despite these limitations, we identified a sufficient number of studies (n = 112) to test our hypothesis examining the association of industry sponsorship, risks of bias and research outcomes for TZDs. A comprehensive inventory of all TZD animal research publications was not necessary in this type of study since we did not seek to report an overall TZD efficacy or harms estimate.

Given that many of the studies included in our meta‐analysis had small samples sizes and often measured multiple outcomes in each animal, we did not account for all reported outcomes to avoid double‐counting animals within studies. Instead, we selected the most common measures reporting changes in glucose, the primary efficacy outcome, and body weight, the primary harms outcome. For example, if a study reported fasting plasma glucose, hepatic glucose output, glucose uptake by tissues and glucose tolerance test results, we included the fasting plasma glucose data and did not account for the other glucose measures. Even though this strategy does not capture all outcomes reported by the investigators in each study, it allowed us to avoid falsely exaggerating effect sizes in our meta‐analysis.

## Conclusions

Non‐disclosure of funding sources or financial ties of investigators was very common in our cohort of animal studies. Risk of bias criteria were poorly reported across studies, regardless of source of funding. The majority of studies had favourable TZD outcomes and conclusions. Industry‐sponsored studies had exaggerated effect sizes for both efficacy and harms in comparison with studies sponsored by non‐industry or a combination of industry and non‐industry sources which could not be explained by methodological differences in the studies. We expect reporting of risk of bias criteria in animal research to improve as risk of bias assessments become more common and as research funders and journals start to adopt and enforce better reporting standards.

## Conflict of Interest

LB, DK and AA have no competing interests to declare. MAS previously worked as a paid summer intern at Genentech and served as a paid Genentech campus ambassador for 2 years at the University of California, San Francisco.
